# Stress and overweight/obesity among nursing students

**DOI:** 10.1590/1518-8345.2966.3177

**Published:** 2019-10-07

**Authors:** Janete de Souza Urbanetto, Pâmela Silva da Rocha, Rosangela Carvalho Dutra, Maria Carolina Maciel, Andrea Gonçalves Bandeira, Tania Solange Bosi de Souza Magnago

**Affiliations:** 1 Pontifícia Universidade Católica do Rio Grande do Sul , Escola de Ciências da Saúde , Porto Alegre , RS , Brasil .; 2 Pontifícia Universidade Católica do Rio Grande do Sul , Hospital São Lucas , Porto Alegre , RS , Brasil .; 3 Hospital Nossa Senhora das Graças , Porto Alegre , RS , Brasil .; 4 Universidade Federal de Santa Maria , Departamento de Enfermagem , Santa Maria , RS , Brasil .

**Keywords:** Students, Nursing, Nursing, Stress, Psychological, Obesity, Overweight, Life Style, Estudantes de Enfermagem, Enfermagem, Estresse Psicológico, Obesidade, Sobrepeso, Estilo de Vida, Estudiantes de Enfermería, Enfermería, Estrés Psicológico, Obesidad, Sobrepeso, Estilo de Vida

## Abstract

**Objective:**

analyze associations between demographic, academic, health, stress, overweight and obesity characteristics among nursing students.

**Method:**

this is a cross-sectional study with 95 students from a private university in Rio Grande do Sul, Brazil. A demographic, academic and health characterization questionnaire and the Assessment of Stress in Nursing Students (ASNS) scale were applied. Anthropometric measures were taken and descriptive and bivariate analyses were performed.

**Results:**

female students predominated in this study, mean age: 25.6±5.87 years. Weight gain was observed in 52.6% of the students, with the ‘Professional training’ session reporting high (29.5%) and very high (36.8%) levels of stress. None of the stress scale sessions was associated with overweight and obesity.

**Conclusion:**

overweight and obesity were associated with male participants, high blood pressure, weight gain since the beginning of the course, altered waist circumference, no physical activity, eating more in stressful situations, and consumption of unhealthy foods.

## Abstract (pt)

**Objetivo:**

analisar a associação entre características demográficas, acadêmicas, de saúde, estresse, sobrepeso e obesidade em estudantes de enfermagem.

**Método:**

estudo transversal, com 95 estudantes de uma universidade privada do Rio Grande do Sul, Brasil. Aplicou-se questionário de caracterização demográfica, acadêmica e de saúde e utilizou-se a Escala de Avaliação do Estresse em Estudantes de Enfermagem. Medidas antropométricas foram mensuradas. Empregaram-se análises descritivas e bivariadas.

**Resultados:**

predominaram estudantes do sexo feminino, com idade média de 25,6 ± 5,87 anos. Observou-se aumento de peso em 52,6% dos estudantes e níveis de estresse alto (29,5%) e muito alto (36,8%) no domínio Formação Profissional. Nenhum dos domínios da escala de estresse se mostrou associado ao sobrepeso e obesidade.

**Conclusão:**

o sobrepeso e obesidade mostraram-se associados ao sexo masculino, pressão arterial elevada, ganho de peso desde o início do curso, circunferência abdominal alterada, não realização de atividade física, comer mais em situações de estresse e ingerir alimentos não saudáveis.

## Abstract (es)

**Objetivo:**

analizar la asociación entre las características demográficas, las académicas, las de salud, el estrés, el sobrepeso y la obesidad en estudiantes de enfermería.

**Método:**

el estudio transversal, con 95 estudiantes de una universidad privada, Rio Grande do Sul, Brasil. Se han aplicado el cuestionario de caracterización demográfica, académica, salud y la escala Evaluación de Estrés en Estudiantes de Enfermería. Las medidas antropométricas han sido mensuradas. Se emplean los análisis descriptivos y los bivariados.

**Resultados:**

han predominado estudiantes del sexo femenino, con edad media de 25,6 ± 5,87 años. Se ha observado el incremento de peso en el 52,6% de los estudiantes y los niveles de estrés alto (el 29,5%) y muy alto (el 36,8%) en el dominio Formación profesional. Ninguno de los dominios de la escala de estrés se ha mostrado asociado al sobrepeso y a la obesidad.

**Conclusión:**

el sobrepeso y la obesidad se han mostrado asociados al sexo masculino, a la presión arterial elevada, a la ganancia de peso desde el inicio del curso, a la circunferencia abdominal alterada, a la no realización de actividad física, al comer más en situaciones de estrés y al ingerir alimentos que no son sanos.

## Introduction

In the last decades, obesity has been related to social and behavioral changes, consumption of caloric foods, high palatability and low satiety power, as well as higher caloric content of every meal and meals outside home in fast food chains ^1^ . It is considered an aggravation of multi-factor character, involving biological, historical, ecological, economic, social, cultural and political issues ^2^ .

According to the Brazilian Association for Obesity and Metabolic Syndrome Studies (ABESO), psychological stress can contribute to weight gain and obesity, as anxiety can lead to increased food intake, inadequate appetite control, and compulsive overeating ^1^ . It is also a complex aggravation due to its multiple causes and occurs in the interaction between an individual and the environment ^3 - 4^ . This mutual influence can cause physical, psychic, emotional and behavioral changes ^4^ . A study that evaluated the night eating syndrome showed that students from a Brazilian university present a high prevalence of this behavior, which has been associated with depression symptoms, anxiety and stress ^5^ .

In daily life, stress is detected through observable behaviors ^3^ . Stressors may arise at different moments due to pressure related to personal, social, professional, and academic life. In the latter, changes, fears and distress, among other developments, can act as stress triggers and aggravate health issues ^6^ .

Nursing students consider the fear of the unknown, overload of academic activities, feeling insecure and powerless ^7^ , dissatisfaction with the course, and excessive activities ^8^ as stressors, as well as increased responsibility in the last year of the course; they also reported that developing the end-of-course assignment, concerns about insertion in the labor market, the work/study/family life relationship, and challenges in interpersonal relationships are situations that cause physical exhaustion and distress ^9^ .

In this context, the demands of academic life and the uncertainty related to the professional career may cause an important emotional load to students, changing their behaviors and lifestyle. According to health students, academic activities, besides causing stress, encourage students to consume food out of the home ^10^ . In the medium and long time, without a balanced diet, this behavior can contribute to weight gain among students.

Considering the above, this theme should be further explored to help identify the factors related to overweight/obesity among university students and support actions to cope with this situation. The study questions were: What are the factors associated with overweight or obesity among nursing students? Is stress associated with overweight or obesity among nursing students? To answer these questions, the following objective was defined: analyze associations between demographic, academic, health, stress, overweight and obesity characteristics among nursing students.

## Method

This is a cross-sectional study that is part of a cohort study project titled *Avaliação da Ocorrência de Dor Musculoesquelética e Distúrbios Psíquicos Menores em Estudantes de Enfermagem* (Assessment of Musculoskeletal Pain and Minor Psychiatric Disorders among Nursing Students – free translation). The study was conducted in a private university in Rio Grande do Sul, Brazil, which offers nursing programs.

The study population had 144 students from the nursing course mentioned above. For sample calculation, this study considered an estimated percentage of 0.5%, 95% confidence interval, and sample error of 0.05%. Based on this calculation, to ensure a representative sample, the minimum number of participants would be 89 nursing students. However, all 114 students were invited to participate in the study. The inclusion criteria were: students should be regularly enrolled in the nursing course, aged ≥18 years. Students away from academic activities due to medical leave, mobility, temporary course interruption, or for any other reason, were not included in the study.

Data collection was performed between September and December 2016, using non-probability sampling (all of them were invited), as scheduled with the students. The variables collected were: a) Demographic characteristics: sex (female, male), age (in years, and later, they were dichotomized as ≤26 years and ≥27 years), marital status (single, married/stable union), skin color (white, black, brown), with children (yes, no); b) Academic characteristics: course semester and start year; c) Health: weight (kg), height (cm), waist circumference (cm), blood pressure (mmHg), physical activity (no, yes, sometimes), and questions assessing the eating habits of students ^11^ ; d) Stress of the students, assessed through the scale of Assessment of Stress in Nursing Students (ASNS) ^3^ .

The ASNS scale has 30 questions that assess the level of stress. Each question has 4 options of answer (0 = I do not experience this situation; 1 = I do not feel stressed with this situation; 2 = I feel a little stressed with this situation; 3 = I feel very stressed with this situation). The ASNS has six sessions: Practical activities; Professional communication; Time management; Environment; Professional training; and Theoretical activities ^3^ .

Session 1 ‘Practical activities’ consists of six items that refer to the instrumental knowledge acquired by the student to perform the procedures, and the feelings involved in the provision of care to patients. In Session 2 ‘Professional communication’ the four items address the challenges experienced in communication and relationship of the individual with the elements of professional life and its situations of conflict. Session 3 ‘Time management’ has five items, which address challenges reported by the students to combine the academic activities with personal, emotional and social demands. The four items of Session 4 ‘Environment’ address the challenges found when accessing the fields of internship or university and the situations of exhaustion perceived by students due to the transportation system used by them. The six items of Session 5 ‘Professional training’ refer to the student’s concern about the knowledge acquired during the academic training and its impact on his/her future professional life. It also addresses the perception of situations that may occur in his/her professional life. The five items of Session 6 ‘Theoretical activities’ address the challenges facing students related to the nursing program content, the activities performed by them and the program methodology adopted by the institution ^3^ .

The levels of stress were classified by session, by adding the scores of the questions from each session: Session 1: 0-9 low level of stress; 10-12 medium level of stress; 13-14 high level of stress; 15-18 very high level of stress. Session 2: 0-5 low level of stress; 6 medium level of stress; 7-8 high level of stress; 9-12 very high level of stress. Session 3: 0-10 low level of stress; 11-12 medium level of stress; 13-14 high level of stress; 15 very high level of stress. Session 4: 0-7 low level of stress; 8-10 medium level of stress; 11 high level of stress; 12 very high level of stress. Session 5: 0-9 low level of stress; 10 medium level of stress; 11-12 high level of stress; 13-18 very high level of stress. Session 6: 0-9 low level of stress; 10-11 medium level of stress; 12-13 high level of stress; 14-15 very high level of stress ^3^ .

Blood pressure (BP) was measured twice according to the 7 ^th^ Brazilian Guideline of Arterial Hypertension, with the arm supported at the height of the heart and the palm of the hand facing up, making sure the clothes were not pressing the limb, using a proper cuff size for the arm circumference; the student was sitting with uncrossed legs, feet resting on the floor, back resting on the chair and relaxed ^12^ .

The first measurement was performed with the student resting for 5 to 10 minutes in a calm environment, making sure the student had no full bladder, not exercised for at least 60 minutes, not consumed alcohol, coffee or food or smoked 30 minutes before the measurement. The second measurement was performed after completing the questionnaire, around 20 minutes between the first and second measurements. The measurement equipment was digital, certified, well calibrated and maintained, and transported properly.

For BP classification, the recommendation of the 7 ^th^ Brazilian Guideline of Arterial Hypertension was used, so students were considered hypertensive with systolic BP (SBP) ≥140mmHg and/or diastolic BP (DBP) ≥90mmHg, according to the mean values of the first and second measurements ^12^ .

Weight at the beginning of the nursing course was self-reported and the current weight was measured using a digital scale. The difference between them was dichotomized by the median as a function of the abnormal distribution (Shapiro-Wilk = 0.022). Height was measured with a stadiometer. For the calculation of body mass index (BMI), BMI=weight/height ^2^ was applied. For BMI classification, cut-off points of 18.5-24.9 kg/m ^2^ (good nutrition), 25-29.9 kg/m ^2^ (overweight) and ≥30 kg/m ^2^ (obesity) were used ^1 , 13 , 14^ .

For central obesity assessment, waist circumference was measured by placing the tape measure at the midpoint between the lower costal edge and the upper edge of the iliac crest, with the participant in the orthostatic position. Considering the cut-off points for waist circumference (90 cm for male and 80 cm for female participants) and the ethnical origin recommended by the International Diabetes Federation ^15^ and the 7 ^th^ Brazilian Guideline of Arterial Hypertension ^12^ , cardiovascular risks were categorized as follows: no risk (<90 cm waist circumference for male and <80 cm for female participants), increased risk (≥90 to <102 cm for male and ≥80 to <88 cm for female participants) and significantly increased risk (≥102 cm for male and ≥88 cm for female participants) ^14^ .

After double entry of data in an Excel spreadsheet and correction of typing inconsistencies, data were analyzed in PASW Statistics® (Predictive Analytics Software, of SPSS Inc., Chicago, USA) 18.0 for Windows, using descriptive and inferential statistics. Quantitative data were described in measures of central tendency (mean or median) and dispersion (standard deviation, amplitude) according to their distribution of normality or not (Shapiro-Wilk test). Associations between independent variables (demographic, academic data, habits and health) and outcomes (overweight and obesity) were measured by the chi-squared test or Fisher’s exact test (less than 5 cells). In all analyses, the significance level of 5% was considered.

The study project was approved by the Ethics Committee of the institution under registration CAAE 50096615.1.0000.5336. All students who participated in the study signed an informed consent form in two counterparts.

## Results

This study had the participation of 95 students (83.3%) from all semesters of the nursing course of a private university in Rio Grande do Sul, Brazil. Of these, 34 (35.8%) students were from semester 8, 21 (22.1%) from semester 6, 18 (18.9%) from semester 2, and 17 (17.9%) from semester 4 of the course; 19 students (16.7%) did not agree to participate in the study.

Table 1 shows the classification of students regarding their demographic profile, physical activity, body mass index, blood pressure levels and waist circumference.

**Table 1:** Characterization of demographic and health variables of nursing students. Porto Alegre, RS, Brazil, 2016. n=95 students

Variables	n	%
Age		
Up to 26 years	64	67.4
27 years old or more	31	32.6
Sex		
Female	76	80.0
Male	19	20.0
Self-reported skin color (n=94)		
White	85	90.4
Black	6	6.4
Brown	3	3.2
Marital status (n=93)		
Single	74	79.6
Married/stable union	19	20.4
Has children (n=90)		
Yes	23	25.5
No	67	74.4
Physical activity		
Yes	17	17.9
No	43	45.3
Sometimes	35	36.8
Body mass index		
Good nutrition	45	47.4
Overweight	34	35.8
Obesity I	11	11.6
Obesity II	5	5.2
Blood pressure classification		
Normal	29	30.5
Pre-hypertension	42	44.2
Hypertension I	14	14.7
Hypertension II	7	7.4
Hypertension III	3	3.2
Waist circumference (cardiovascular risk - sex)		
<80 cm (no risk - female)	38	40.0
<90 cm (no risk - male)	7	7.4
≥80 to <88 cm (increased risk - female)	19	20.0
≥90 to <102 cm (increased risk - male)	10	10.5
≥88 cm (significantly increased risk - female)	19	20.0
≥102 cm (significantly increased risk - female)	2	2.1

Source = Study data, 2016

This study had a prevalence of students aged up to 26 years (25.68±5.87 years, min. 18 years and max. 43 years), female, self-reported white, single, without children, and who did not practice physical activity. A higher percentage of students had weight issues (overweight, obesity I and II), were classified as pre-hypertensive, with cardiovascular risk ( Table 1 ).

Regarding the self-reported weight at the beginning of the course, mean weight was 68.08 kg. Mean current weight was 71.43 kg. Considering the difference between the two values, half of the students had a gain of 3.55 kg from the beginning of the course to the study moment. The minimum weight loss was 42.3 kg and maximum gain was 28 kg.

When asked if they considered their diet as healthy, 55 (57.9%) answered no, and 42 (44.2%) students said that replaced their main meals of the day with fast snacks. Regarding the traditional meals of the day, 38 (40%) students eat breakfast, 52 (54.7%) eat a morning snack, 90 (94.6%) eat lunch, 69 (72.6%) at an afternoon snack, 92 (86.3%) eat dinner and 21 (22.1%) eat supper. A higher percentage of students (74.7%) usually eat meals watching TV, using their computer, tablet and/or cell phone. In situations of stress, 63 (66.3%) students answered they tend to eat more.

Table 2 presents the stress classification (low, medium, high, very high level of stress), according to the ASNS scale sessions.

**Table 2:** – Stress classification according to each session of the scale of Assessment of Stress in Nursing Students (ASNS). Porto Alegre, RS, Brazil, 2016. n=95 students

Categories of stress	Sessions of the ASNS scale*					

	Practical activities	Professional communication	Time management	Environment	Professional training	Theoretical activities

	n (%)	n (%)	n (%)	n (%)	n (%)	n (%)
Low level of stress	31(32.6)	41(43.2)	60(63.2)	58(61.1)	23(24.2)	42(44.2)
Medium level of stress	30(31.6)	23(24.2)	11(11.6)	17(17.9)	9(9.5)	27(28.4)
High level of stress	22(23.2)	17(17.9)	18(18.9)	8(8.4)	28(29.5)	16(16.8)
Very high level of stress	12(12.6)	14(14.7)	6(6.3)	12(12.6)	35(36.8)	10(10.5)

Source = Study data, 2016. *ASNS - Assessment of Stress in Nursing Students

Table 3 shows data of BMI associated with demographic, academic, labor and health variables.

**Table 3:** Association of demographic, academic, labor and health variables with body mass index. Porto Alegre, RS, Brazil, 2016. n=95 students

Independent variables	Body mass index*						p

	Good nutrition		Overweight		Obesity		

	n	%	n	%	n	%	
Age							
Up to 26 years	34	53.1	23	35.9	7	10.9	0.066 ^†^
27 years old or more	11	35.5	11	35.5	9	29.0	
Sex							
Female	40	52.6	22	28.9	14	18.4	0.032 ^‡^
Male	5	26.3	12	63.2	2	10.5	
Weight variation (n=90)							
Weight variation ≤3.55 kg	32	71.1	9	20.0	4	8.9	<0.001 ^‡^
Weight variation ≥3.55 kg	12	26.7	21	46.7	12	26.7	
Waist circumference							
No risk	36	80.0	9	20.0	-	-	<0.001 ^†^
Increased risk	8	27.6	20	69.0	1	3.7	
Significantly increased risk	1	4.8	5	23.8	15	71.4	
Physical activity							
Sometimes	16	45.7	16	45.7	3	8.6	0.027 ^‡^
Yes	7	41.2	9	52.9	1	5.9	
No	22	51.2	9	20.9	12	27.9	
Blood pressure							
Normal	16	55.2	8	27.6	5	17.2	0.042 ^†^
Pre-hypertension	24	57.1	13	31.0	5	11.9	
Hypertension	5	20.8	13	54.2	6	25.0	
Course semester							
Up to semester 4	19	51.4	12	32.4	6	16.2	0.616 ^†^
Semester 5 to 8	26	44.8	22	37.9	10	17.2	
Meal replaced with snacks (n=93)							
Yes	15	35.7	20	47.6	7	16.7	0.139 ^†^
No	29	56.9	13	25.5	9	17.6	
Reaction in situations of stress or anxiety							
Tend to eat more	24	38.1	25	39.7	14	22.2	0.025 ^‡^
Tend to eat less	21	65.6	9	28.1	2	6.3	
Food intake							
Consider consumed food as healthy	20	36.4	24	43.6	11	20.0	0.042 ^†^
Do not consider consumed food as healthy	25	62.5	10	25.0	05	12.5	

Source = Study data, 2016. *Cut-off points for body mass index = 18.5-24.9 kg/m ^2^ (good nutrition), 25-29.9 kg/m ^2^ (overweight) and ≥30kg/m ^2^ (obesity); ^†^ Pearson’s chi-squared test; ^‡^ Fisher’s exact test

Table 4 shows data of BMI associated with the ASNS scale sessions.

**Table 4:** – Association of ASNS (Assessment of Stress in Nursing Students) scale sessions with BMI. Porto Alegre, RS, Brazil, 2016. n=95 students

ASNS scale sessions ^*^	Body mass index ^†^						p ^‡^

	Good nutrition		Overweight		Obesity		

	n	%	n	%	n	%	
Practical activities							
Low level of stress	14	45.2	11	35.5	6	19.4	0.680
Medium level of stress	17	56.7	9	30.0	4	13.3	
High level of stress	10	45.5	7	31.8	5	22.7	
Very high level of stress	4	33.3	7	58.3	1	8.3	
Professional communication							
Low level of stress	22	53.7	10	24.4	9	22.0	0.341
Medium level of stress	9	39.1	12	52.2	2	8.7	
High level of stress	7	41.2	6	35.3	4	23.5	
Very high level of stress	7	50.0	6	42.9	1	7.1	
Time management							
Low level of stress	30	50.0	17	28.3	13	21.7	0.378
Medium level of stress	5	45.5	6	54.5	0	0	
High level of stress	7	38.9	9	50.0	2	11.1	
Very high level of stress	3	50.0	2	33.3	1	16.7	
Environment							
Low level of stress	26	44.8	24	41.4	8	13.8	0.674
Medium level of stress	8	47.1	6	35.3	3	17.6	
High level of stress	5	62.5	1	12.5	2	25.0	
Very high level of stress	6	50.0	3	25.0	3	25.0	
Professional training							
Low level of stress	11	47.88	8	34.8	4	17.4	0.659
Medium level of stress	6	66.7	2	22.2	1	11.1	
High level of stress	13	46.4	8	28.6	7	25.0	
Very high level of stress	15	42.9	16	45.7	4	11.4	
Theoretical activities							
Low level of stress	22	52.4	14	33.3	6	14.3	0.465
Medium level of stress	14	51.9	7	25.9	6	22.2	
High level of stress	7	43.8	7	43.8	2	12.5	
Very high level of stress	2	20.0	6	60.0	2	20.0	

Source = Study data, 2016. *ASNS: Assessment of Stress in Nursing Students; ^†^ Cut-off points for body mass index = 18.5-24.9 kg/m ^2^ (good nutrition), 25-29.9 kg/m ^2^ (overweight) and ≥30kg/m ^2^ (obesity); ^‡^ Fisher’s exact test

## Discussion

The study participants were predominantly female nursing students, in agreement with Brazilian studies conducted by the Federal Nursing Council, showing prevalence of female nursing professionals (87.2%) ^16^ . They also agree with other findings in the literature regarding age: young adult (65.5%) and single students (88.6%) ^17^ , and self-reported white skin color (78.9%) ^18^ .

More than half of the study participants report they eat the main meals of the day and do not replace them with quick snacks. The most often meals are lunch and dinner. However, less than half of the students have breakfast and supper. Lunch was also the most frequent meal of a study on the lifestyle of nursing students, who reported it four to seven times a week (73.4%) ^19^ . In this study, students also reported snacks one to three times (45.8%) and four to seven times (24.5%) a week ^19^ .

The habit of performing other activities (for example, watching TV, using cell phone) while eating meals seems recurrent in the literature. The use of cell phone during meals was reported by 14% of the students, due to the need to remain connected (39.5%) ^20^ . This is a reason for concern, as the students, when doing other things while eating, do not have a quiet moment to enjoy the food, eat fast, with impact on satiation, not fulfilling their nutritional needs.

Although the number of students with good nutrition is larger (47.4%), weight changes is a concern when the percentages of overweight (35.8%) and obesity I and II (16.8%) are added together, as they reach 52.6% of the students in this study. These findings exceed the prevalence already identified, where 26% of nursing students from a university in Bahia were overweight and 4.5% presented obesity I and II ^21^ , and combined prevalence of overweight/obesity was 32.4% of health students from two Mexican universities ^22^ .

The 7th Brazilian Guideline of Arterial Hypertension(12) emphasizes that weight gain is directly related to increased BP. In this study, almost 50% of the students had pre-hypertension and 26.3% had hypertension. The changes in waist circumference indicate visceral fat deposition, which is another important factor to be considered in the prevention of health aggravation among nursing students.

Stressful situations can contribute to changes in eating habits, including increased food intake, which predisposes to overweight and obesity in a significant way ^22^ . The students in this study presented high (29.5%) and very high levels (36.8%) of stress, regarding Session 5 of the ASNS, which assesses professional training issues. Other Brazilian studies also showed greater percentages (around 50%) of stress in this session ^23 - 24^ . Several factors may be related, including concern about increasingly competitive labor market, requiring health professionals to attend specialization courses and acquire theoretical-practical knowledge and experience.

Unlike other studies showing a significant relationship among stress, overweight and obesity ^5 , 22^ , the findings of this study did not show statistically significant differences between weight changes of students, according to the BMI classification and the levels of stress evaluated by the ASNS session (p>0.05). Significant relationships were found with some demographic and health variables.

A study that evaluated risk factors for cardiovascular diseases among nursing students associated BMI ≥25 cm in the age groups 21-30 years ^25^ , unlike this study, which did not show any significant difference between the groups evaluated (p=0.06).

Male participants were significantly associated with BMI in overweight (p=0.03) when compared to female participants, who were classified in the ‘good nutrition’ category. Among Mexican students, the prevalence of overweight/obesity was also significantly higher among male students (38.5%) than among female students (30.5%) ^22^ , unlike the Brazilian reality, according to the ABESO website, which reports a higher prevalence of overweight among female individuals (58.2% versus 55.6%) ^26^ , but this difference is very small. Weight variation presented a significant association with BMI (p<0.001); that is, students with ≤3.55 kg weight variation were classified as presenting good nutrition (71.1%) and those with ≥3.55 kg weight variation were classified as presenting overweight (46.7%) and obesity (26.7%).

Waist circumference was associated with BMI (p<0.001); that is, a higher percentage of students with increased cardiovascular risk were classified as presenting overweight (69%) and students with significantly increased risk were classified as presenting obesity (71.4%). In this regard, there is no consensus in the literature, since a study comparing waist circumference and BMI between the first and last year of the nursing course did not find a significant variation of these indicators; however, 59.1% of the students did not have proper waist circumference ^21^ , which is close to the percentage found in this study (52.6%).

These findings are supported by the fact that students do not consider their eating habits as healthy and do not usually practice physical activity. Those who do not practice physical activity were classified as presenting obesity (27.9%). According to ABESO, modern lifestyle favors weight gain. Eating fast and using cell phone during meals, for example, are obstacles to satiation. Reduced sport practice and lower daily energy expenditure are also mechanisms that influence weight gain, which may be related to lack of time ^2^ and the dynamics of academic life. A study evaluating the practice of physical activity among nursing students reported 57.8% of the students were sedentary ^27^ . The 7 ^th^ Brazilian Guideline of Arterial Hypertension reports physical inactivity has been considered one of the major public health problems, due to its high prevalence, and because it is the second main cause of death in the world ^12^ .

The physical consequences of physical inactivity can happen sequentially, since the students classified with hypertension had greater distribution in the categories of overweight (54.2%) and obesity (25%). According to ABESO, weight reduction also lowers blood pressure and improves the levels of HDL (high density lipoprotein) and LDL (low density lipoprotein), blood sugar, among others. Changes in behavior can reduce blood pressure and the risk of hypertension in one year ^1^ .

A healthy lifestyle also helps reduce anxiety in situations of stress, which are harmful and relevant in the relationship between eating more and being overweight (39.7%) when compared to those classified in the ‘good nutrition’ category, and even in situations of stress, they do not feel like eating more (65.6%; p=0.025). These results may be linked with an association between feeling stress and the feeling better after eating palatable foods, with sugar and fat. This behavior may become a habit and people can start using food to relieve stress ^1^ . Increased consumption of foods in situations of anxiety is frequently reported in the literature ^5 , 22^ .

The study limitations were: cross-sectional design (reverse causality bias); self-reported body weight at the beginning of nursing course (no data had been previously collected on this variable); the scarce literature that associates factors related to stress (ASNS scale), overweight and obesity among nursing students. One challenge with the ASNS scale is that it does not have a general cut-off point to classify students in a situation of stress, since classification is by ASNS session. Another challenge referred to student adherence to the study, a fact related to class schedules, lack of time, reported embarrassment due to values of anthropometric measurements, especially weight. However, the minimum sample size was obtained for representative findings of the nursing student population.

## Conclusions

The population of this study showed no association among stress, overweight and obesity. A significant association was observed between overweight and/or obesity and male students, high blood pressure, weight gain since the beginning of the course, altered waist circumference, no physical activity, eating more in stressful situations, and eating unhealthy foods.

Despite study limitations and challenges, some stressors were identified for nursing students, which can support the development of strategies to reduce such stressors and contribute to the professional training of nurses and reduced aggravations related to overweight and obesity. This topic should be further studied in future investigations and widely discussed so that students can dedicate attention to their health and the risks that may affect it. As future nurses, they play an important role in promoting health and preventing diseases, especially cardiovascular and psychosomatic diseases. Then, students, since the beginning of the course, also play an important role in promoting their health and preventing aggravations. For this reason, health promotion strategies should be developed among nursing students to improve this scenario and minimize future risks that may result from obesity.
